# Temporal Trends of Candida Species in Healthcare-Associated Infections in Intensive Care Units in Taiwan

**DOI:** 10.3390/medicina62050814

**Published:** 2026-04-24

**Authors:** Chih-Chun Hsiao, Yu-Hsuan Chen, Chun-Gu Cheng, Chun-An Cheng

**Affiliations:** 1 Department of Nursing, Taoyuan Armed Forces General Hospital, Taoyuan 32549, Taiwan; 2 Division of Chest Medicine, Department of Internal Medicine, Cheng Hsin General Hospital, Taipei 11220, Taiwan; anaemia0829@gmail.com; 3 Department of Emergency Medicine, Taoyuan Armed Forces General Hospital, Taoyuan 32549, Taiwan; 4 Department of Emergency Medicine, Tri-Service General Hospital, National Defense Medical University, Taipei 11490, Taiwan; 5 Department of Neurology, Tri-Service General Hospital, National Defense Medical University, Taipei 11490, Taiwan

**Keywords:** Candida, health-associated infection, intensive care unit

## Abstract

*Background and Objectives:* The epidemiological characteristics of Candida species have changed worldwide, with an increasing number of reports on co-infections with non-*albicans* Candida species (NACs) and multidrug-resistant bacteria. A longer length of hospital stay, more severely ill patients, and empirical antimicrobial use in intensive care units (ICUs) increased the prevalence of Candida healthcare-associated infections (HAIs). If the diagnosis or treatment of invasive candidiasis is delayed, the morbidity and mortality of patients will significantly increase. *Materials and Methods*: We conducted a nationwide surveillance study to analyze data on HAIs in the ICUs of medical centers and regional hospitals between 2018 and 2023. We assessed the temporal trends of Candida species (including *Candida albicans* and NACs) across all HAIs, bloodstream infections (BSIs), and urinary tract infections (UTIs), and simultaneously assessed the corresponding trends of *Enterococcus faecium* (Efm). A linear trend for the proportions of microorganisms from 2018 to 2023 was noted according to the Mantel–Haenszel chi-square test. Spearman’s rank correlation coefficients were used to analyze the correlations between pathogen proportions, systemic antimicrobial agent consumption, and length of ICU stay. *Results*: The overall proportion of all Candida species in HAIs in the ICUs increased significantly from 15.13% to 16.74% (*p <* 0.001); this increase was driven mainly by NACs (increasing from 6.84% to 7.91%, *p <* 0.001) from 2018 to 2023. The proportion of Efm increased significantly, from 7.7% to 11.11% (*p <* 0.001). The proportions of all Candida species significantly increased in UTIs (from 24.63% to 28.13%, *p <* 0.001), especially NACs, while the proportion of Efm also increased significantly in UTIs (from 9.47% to 15.32%, *p <* 0.001). With respect to the UTIs, the proportion of all the Candida species, *C.albicans*, and NACs were positively correlated with the amount of systemic antibiotics used. A longer hospital stay was strongly correlated with all Candida HAIs and UTIs, especially NACs. Significantly ecological associations between all the Candida strains and Efm were observed for UTIs. *Conclusions*: This study revealed that a persistent expansion of NAC infections was associated with increased Efm infections and rising antibiotic consumption. The changes in the proportions of different Candida species in UTIs were most pronounced. These findings support an ecological model in which antibiotic stress and chronic critical illness contribute to the expansion of fungal–bacterial co-infections in the ICU setting and underscore the need for integrated antibiotic management and multi-infection surveillance.

## 1. Introduction

Healthcare-associated infections (HAIs) remain a major threat to patient safety worldwide, leading to longer hospital stays, increased healthcare costs, and significant increases in morbidity and mortality [1,2,3]. The burden of HAIs is particularly pronounced in intensive care units (ICUs), where critically ill patients are exposed to invasive medical devices, broad-spectrum antibiotic treatment, and severe immune dysregulation. Candida was the most common fungal HAI. Invasive candidiasis primarily affects immunocompromised or critically ill patients [4]. In recent years, invasive candidiasis has increased among HAIs during and after the COVID-19 pandemic [5,6].

*Candida albicans* is a common component of the human gut microbiota and a commensal species in the skin and genitourinary system. It is dependent on numerous interactions between different cells between different species of the same genus and pathogen–host interactions [7]. Among various human HAIs, fungal infections, especially those involving Candida species, have shown a gradual upward trend in recent years. Candida spp. is also more likely to colonize and infect severely ill patients because of invasive treatments and broad-spectrum antibiotic use; it is associated with immunodeficiency, such as systemic lupus erythematosus [8]. Higher biofilm production and metabolic activity have been noted in invasive candidiasis [9]. Candida infection increased with increasing mortality during the COVID-19 era [10,11,12]. An older age, ICU stay, more severe disease, and the use of steroids are related to mortality in the COVID-19 era [13]. Non-*albicans* Candida species (NACs) have reduced sensitivity to azole drugs, and resistance to echinocandins has emerged in some regions [14]. Antibiotic use, prolonged ICU stay, bacterial-fungal co-infection are considered important risk factors for Candida infections, but their relationship from nationwide surveillance data in ICUs in Taiwan has not yet been fully explored.

Given the aforementioned knowledge gap, we utilized nationwide surveillance data on HAIs in Taiwan from 2018 to 2023 from the HAI Surveillance System to explore the changes in the proportions of different Candida species in HAIs of ICUs. We attempted to determine the temporal trends of the overall proportion and specific species proportions of Candida in HAIs. We also assessed the associations among Candida epidemiology, systemic antimicrobial drug consumption, length of stay in the ICU, and other bacterial microorganisms.

By integrating nationwide surveillance data and antimicrobial drug use indicators, we aimed to elucidate the broader ecological factors influencing fungal epidemiology in the ICU environment and provide information for population-level antimicrobial drug management and infection prevention strategies. Moreover, we also examined the potential association between Candida and bacterial infection to enhance the understanding of the co-changing trends of fungi and bacteria. Through this analysis, we aimed to provide empirical references for clinical and infection control personnel in antimicrobial management, ICU infection prevention and control, and fungal infection surveillance.

## 2. Materials and Methods

Since 2007, the HAI Surveillance System has published an annual report on HAIs reported in ICUs of medical centers and regional hospitals in the Taiwan Centers for Disease Control (CDC). The reports of bacterial or fungal culture provided by participated hospitals reported through web-based entry. Surveillance data adopting voluntary reporting included HAI cases reported in the ICUs of 24 general hospitals (MCs) and 82 regional hospitals (RHs) since 2022; the participating hospitals showed no change in the study period from 2018 to 2023. The definition of an HAI followed that of the Taiwan CDC. HAI generally refers to infections that patients acquire while receiving medical or surgical treatments developed in a hospital or other healthcare facilities that first appear 48 h or more after hospital admission, or within 30 days after having received healthcare [15].

**Figure 1:**
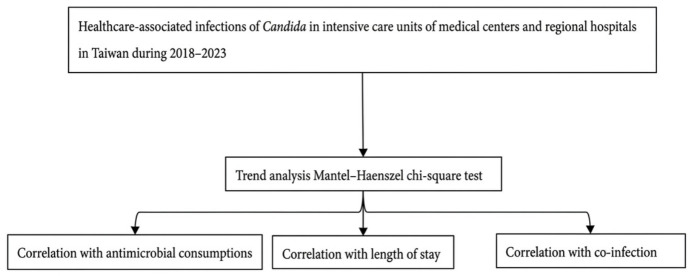
The flowchart of this study.

HAIs are defined on the basis of the type of infection, including bloodstream infections (BSIs), urinary tract infections (UTIs), hospital-acquired pneumonia, surgical site infections, and infections in other parts of the body [15]. We analyzed antibiotic consumption during hospitalization in Taiwan from 2018 to 2023, and considered the Anatomical Therapeutic Chemistry Classification (ATC) code J01 for all antibiotics and J01D for other beta-lactam antibiotics [16]. We also collected the mean length of hospital stay in the ICU [17]. This study was approved by the Ethics Committee of Tri-Service General Hospital, approval number TSGH-C202305039. The flowchart is shown in Figure 1.

The linear trend of the proportions of Candida and bacteria from 2018 to 2023 was analyzed using the Mantel–Haenszel chi-square test. The minimal clinically important difference (MCID) was estimated using a distribution-based method. Spearman’s correlation coefficients were used to analyze the correlations between different types of Candida and antibiotic consumption or length of hospital stay. The changes in microorganisms were compared between 2018 and 2023 using crude odds ratios (ORs) for Candida and other bacteria. The relative risk of antimicrobial consumption, length of ICU stays, and bacterial microorganisms in Candida HAIs were analyzed with general linear regression. Statistical analyses were performed using SPSS version 21 (Asia Analytics, Taipei, Taiwan).

## 3. Results

**Figure 2:**
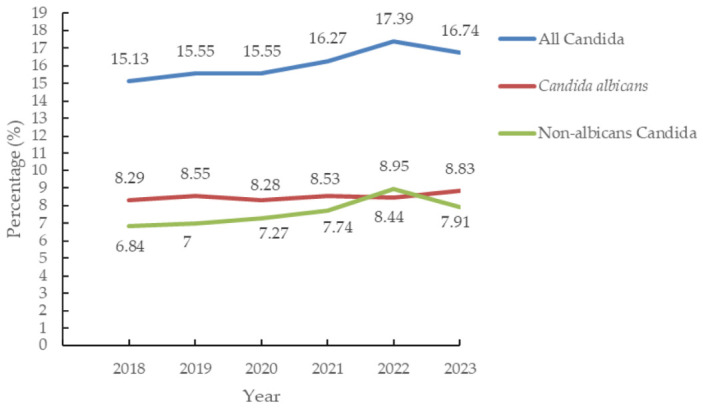
The trends of candida in healthcare-associated infectionsin intensive care units in Taiwan.

The proportion of all Candida species increased from 15.13%to 16.74% (*p <* 0.001), with an OR of 1.128 (95% confidence interval (C.I.): 1.048–1.215; *p* = 0.001) in 2023 compared with that in 2018. The proportion of NACs increased from 6.84% to 7.91% (*p <* 0.001), with an OR of 1.17 (95% C.I.:1.055–1.299, *p* = 0.003) in 2023 compared with 2018; and the proportion of *Enterococcus faecium* (Efm) increased from 7.7% to 11.11% (*p <* 0.001), with an OR of 1.498 (95% C.I.: 1.363–1.647, *p <* 0.001) (Table 1) (Figure 2).The MCID of annual proportion changes in total Candida HAI was 0.347%, *Candida albicans* HAI was 0.14%, and NAC HAI was 0.4% (Supplementary Table S1 shows the temporal change in the proportion of HAIs attributable to Candida species).

With respect to UTIs, the proportion of all Candida species increased from 24.63%to 28.13% (*p <* 0.001), the proportion of NACs increased from 10.13%to 11.96% (*p <* 0.001), and the proportion of Efm changed from 9.47% to 15.32% (*p <* 0.001) (Table 2).

The proportions of NACs and all Candida species in HAIs and UTIs were positively correlated with antibiotic consumption; *Candida albicans* in UTIs was positively correlated with antibiotic consumption (Table 3). NACs and all Candida species were positively correlated with the length of ICU stay among patients with HAIs or UTIs (Table 3).

All Candida species and NACs were positively correlated with Efm in both HAIs and UTIs. *Candida albicans* in UTIs was positively correlated with Efm in UTIs (Table 4). The RR of NACs in HAIs showed an adjusted RR of 1.048 (95% C.I.: 1.036–1.061, *p <* 0.001) for length of ICU stay and adjusted RR of 10.545 (95% C.I.: 2.665–41.726, *p* = 0.001) for Efm. The RR of NACs in UTIs showed an adjusted RR of 1.045 (95% C.I.: 1.033–1.056, *p <* 0.001) for length of ICU stay and adjusted RR of 7.347 (95% C.I.: 1.522–35.465, *p* = 0.013) for Efm of UTIs (Table 5).

## 4. Discussion

In this nationwide surveillance analysis covering the period from 2018 to 2023, we reported a significant increase in the proportion of patients with Candida HAIs, predominantly NACs, in the ICUs of Taiwan. This upward trend is associated with increased antibiotic consumption, prolonged ICU stays, and a corresponding increase in the prevalence of Efm. In conclusion, our findings suggest that the epidemiology of Candida in ICUs is not an isolated fungal phenomenon but rather reflects a broader ecological disturbance related to antibiotic stress and the device-associated biofilm environment.

### 4.1. Epidemiological Trends Shift to Non-Albicans Candida Species

While *C. albicans* remains the dominant pathogen, our data show a disproportionate increase in the infection rate of HAIs with NACs. *C. glabrata* and *C. parapsilosis* are common in Western countries. In the Asia-Pacific region, antifungal drug resistance to Candida is widespread and increasing [18]. *C. tropicalis* is the second most virulent Candida species and can also form true hyphae and complex biofilms in vitro and secrete proteases, phospholipases, and hemolysins [19]. *C. tropicalis* is related to one-month mortality with an odds ratio (OR) of 2 in patients with NAC infection [20]. It exhibits reduced susceptibility to azoles, and the resistance to fluconazole increased from 8.5% to 10.8% in *C. tropicalis* from 2014 to 2022 in Taiwan [21]. There is a higher risk of mortality with *C. tropicalis* in patients with NACs [20]. *C. tropicalis* is the most common species isolated from the surface of fruits in Taiwan, and approximately 18.6% were resistant to fluconazole from the environment [22]. Evidence suggests that fungal resistance likely develops after exposure to azole fungicides in environmental or agricultural settings [23]. The standard dosage of antifungal drugs does not consider pharmacokinetic differences in critically ill patients, which may lead to insufficient drug exposure [18]. Our findings extend these concerns regarding the increase in NACs to the national level in the ICU setting and correlation with antifungal use. But the special species of NACs and azole resistance were not supported by annual survey data that do not fully consider the influence of different NACs in HAIs. The delay in treatment worsens outcomes; NAC therapy is more expensive because of the need to use of echinocandins. However it has a higher cost-effectiveness becuause it improve the prognosis [18].

### 4.2. Antimicrobial Stress as a Driver of Fungal Proliferation

A key finding of this study is the positive correlation between systemic antimicrobial consumption and the prevalence of *C. albicans* and NACs in UTIs. These findings support the ecological hypothesis that broad-spectrum antimicrobial therapy disrupts symbiotic microbiota, leading to fungal overgrowth and mucosal invasion. Researchers have confirms that antibiotics impaired antifungal immunity and induced dysbiosis of the microbiome, promoting fungal translocation and organ dissemination [24,25]. Traditional antimicrobial management indicators primarily target bacterial treatment; however, our findings suggest that antibiotic intensity may indirectly influence fungal epidemiology within the ICU ecosystem.

### 4.3. Candida–Enterococcus Ecological Convergence

Bacteria related to invasive Candida infection [26]. Mixed BSIs involving Candida/bacteria accounted for 25.2% of all Candidemia cases with higher mortality [27]. Notably, in animal models, antibiotic stress-induced proliferation of Enterococcus was associated with enhanced fungal dissemination [28]. The mortality rate associated with co-infection with vancomycin-resistant enterococci is significantly higher than that associated with Candidemia alone [27]. The synchronous growth of Candida and *Enterococcus faecalis* warrants special attention. Enterococcus is a major multidrug-resistant hospital-acquired pathogen that often cocolonizes mucosal surfaces with Candida. Mechanistic studies of *Enterococcus faecalis* have shown that virulence factors such as gelatinase (GelE), serine protease (SprE), and enterococcal surface proteins (Esps) can increase biofilm formation and tissue invasion [29]. Candida/bacteria mixed infections show higher rates of septic shock, prolonged ICU stays, longer mechanical ventilation, and potentially higher mortality [30]. Septic shock in Candidemia increased mortality [31].

The prevalence of NACs and Efm increased synchronously over time, suggesting fungal–bacterial synergy in high-risk healthcare settings. Although the decrease in the VREfm was associated with infection prevention protocols during the COVID-19 era, Efm has increased in recent years, with more than sixty percent of VRE remaining. Candida should engage participants in a multimicrobial pathogenic network.

### 4.4. Device-Associated Multimicrobial Biofilms and UTIs

A significant increase in the proportion of Candida species has been observed among UTIs, and the proportion of catheter-associated infections (CAUTIs) remains persistent at approximately 89% in Taiwan [15]. Indwelling catheters provide an ideal substrate for the formation of multimicrobial biofilms. The pathogenesis of CAUTIs involves the formation of biofilms on the catheter surface, which hinders standard antimicrobial therapy [32]. Under shear stress conditions, multimicrobial communities can maintain structural integrity and increase metabolic activity. Common Gram-negative pathogens associated with CAUTIs frequently coexist with Candida species in biofilms [33]. This multimicrobial structure may lead to antibiotic resistance and persistent colonization, thereby increasing the risk of secondary BSIs. Foley catheter use was related to one-month mortality with an OR of 1.7 in a patient with an NAC infection [20]. Therefore, our findings show that NACs increased in UTIs and highlight the need for proactive device management strategies, early catheter removal, and comprehensive fungal surveillance in CAUTI prevention programs.

### 4.5. Relationship Between Candidiasis and Hospital Stay

Compared with patients in Western countries, patients in Asia have a shorter mean duration from ICU admission to the onset of Candidemia [34]. Patients infected with Candida have a longer duration of hospitalization. Patients with severe disease who are hospitalized in ICUs with weakened conditions or bacterial infections need antimicrobial treatment. Our study revealed that Candida infection was correlated with longer hospitalization, indicating that patients had more severe conditions. Healthcare staffs need to quickly manage infections as well as the physical management of ICU patients so that they can then easily transfer these patients to general wards to reduce fungal infections.

This study utilizes nationwide surveillance data provided by the Taiwanese CDC, thus enhancing the representativeness and external validity of this study. The six-year longitudinal study design allows for a robust assessment of sustained time trends. Combining Candida epidemiology with antibiotic consumption and length of stay in the ICU provides a rare population ecology perspective. The simultaneous analysis of Candida and other bacteria offers new insights into the potential fungal–bacterial fusion in an ICU setting.

This study has several limitations. First, the annual database lacks patient-level clinical variables, such as age, disease severity, duration of device use, complications, comorbidities, prior antifungal drug exposure, lack of radiological or microbiological data on fulminant forms and antifungal drug susceptibility profiles, and time of observation, which limits adjustments for potential confounding factors. The surveillance dataset did not include information distinguishing primary from secondary infections, thereby precluding robust inference regarding the causal relationship between bacterial and Candida infections. Second, as an ecological analysis based on nationwide surveillance data from the Taiwanese CDC, causal relationships cannot be established. The observed correlations between antibiotic consumption, duration in the ICU, and increased Candida prevalence may reflect common temporal trends rather than direct causation. Registered studies need to establish the cause–effect relationship. Third, Candida is categorized only as *C. albicans* or NACs and lacks detailed species-level stratification, which limits the interpretation of resistance patterns and clinical significance. *Candida tropicalis* is the dominant species in Taiwan, and resistance to fluconazole has increased in recent years [21]. These factors influence the resistance to antifungal agents of different species and are require an in-depth clinical explaination. Finally, antimicrobial use is analyzed as a summary of the overall dose during hospitalization, preventing the assessment of exposure at the category or individual level. Therefore, specific antibiotic drivers leading to fungal proliferation cannot be identified. Future studies need to analyze antimicrobial consumption at the patient level.

## 5. Conclusions

This nationwide ICU surveillance analysis revealed a sustained increase in the prevalence of Candida (especially NACs) and Efm, which was positively correlated with antibiotic consumption and length of ICU stay. The distributional shifts among Candida species were most prominently observed in UTIs and concurrently associated with a rise in Efm infections. This suggests a transition toward more resistant fungal pathogens and underscores the imperative for coordinated antimicrobial stewardship and enhanced surveillance strategies in ICUs. These findings support a model in which antibiotic-driven ecological disruption, device-associated biofilm formation, and fungal–bacterial synergy reshape pathogen patterns in ICUs. Future research integrating metagenomics, antibiotic resistance phenotyping, and patient antibiotic exposure data is urgently needed to clarify the causal relationships and provide a basis for precise infection control strategies.

## Figures and Tables

**Table 1:** Candida in healthcare-associated infectionsinintensive care units in Taiwan.

	2018	2019	2020	2021	2022	2023	*p*
All Candida	15.13	15.55	15.55	16.27	17.39	16.74	<0.001 *
*Candida albicans*	8.29	8.55	8.28	8.53	8.44	8.83	0.723
Non-*albicans* Candida	6.84	7	7.27	7.74	8.95	7.91	<0.001 *
*Enterococcus faecium*	7.7	8.29	8.25	9	10.08	11.11	<0.001 *

* *p* < 0.05.

**Table 2:** The healthcare-associated infections of Candida and *Enterococcus faecium* in bloodstream infections and urinary tract infections.

	2018	2019	2020	2021	2022	2023	*p*
Bloodstream infections							
*Candida albicans* (%)	5.28	4.94	5.07	5.38	5.09	5.54	0.772
Non-*albicans* Candida (%)	7.08	7.91	7.49	8.34	8.56	7.65	0.105
All Candida (%)	12.46	12.85	12.57	13.72	13.65	13.2	0.35
*Enterococcus faecium*	8.24	8.5	7.77	8.7	10.03	10.68	<0.001 *
Urinary tract infections							
*Candida albicans* (%)	14.51	15.01	15.28	16.22	15.39	16.17	0.518
Non-*albicans* Candida (%)	10.13	9.32	10.31	10.93	13.29	11.96	<0.001 *
All Candida (%)	24.63	24.33	25.59	27.15	28.69	28.13	<0.001 *
*Enterococcus faecium*	9.47	10.15	11.06	12.02	13.06	15.32	<0.001 *

* *p <* 0.05.

**Table 3:** The correlation between different typesof candida and all antimicrobial consumptions during hospitalization or length of hospitalization in intensive care units.

	All Candida	*Candida albicans*	Non-*albicans* Candida	All Candida in BSIs	*Candida albicans* in BSIs	*Non-albicans* Candidain BSIs	All Candida in UTIs	*Candida albicans* in UTIs	Non-*albicans* Candida in UTIs
ATC of J01	0.943	0.486	0.943	0.714	0.371	0.543	0.886	0.829	0.886
*p*	0.005 *	0.329	0.005 *	0.111	0.468	0.266	0.019 *	0.042 *	0.019 *
Length of stay	0.943	0.143	0.943	0.657	0.429	0.543	1	0.714	1
*p*	0.005 *	0.787	0.005 *	0.156	0.397	0.266	<0.001 *	0.111	<0.001 *

* *p <* 0.05; ATC: Anatomical Therapeutic Chemical Code; BSIs: bloodstream infections; UTIs: urinary tract infections. The correlation test between the ATC of J01D and different types of Candida healthcare-associated infections is the same as the ATC of J01 antimicrobials.

**Table 4:** The correlation between different types of Candida and *Enterococcus faecium* in healthcare-associated infections.

	Healthcare-Associated Infections	Urinary Tract Infections	Bloodstream Infections
*Candida albicans*	0.714	0.829	0.486
*p*	0.111	0.042 *	0.329
Non-*albicans* Candida	0.886	0.886	0.6
*p*	0.019 *	0.019 *	0.208
All Candida	0.886	0.886	0.714
*p*	0.019 *	0.019 *	0.111

* *p <* 0.05.

**Table 5:** The relative risk of parameters in non-*albicans* Candida in healthcare-associated infections and urinary tract infections.

	Unadjusted Relative Risk	*p*	Adjusted Relative Risk	*p*
Non-*albicans* Candida in HAIs				
Antimicrobial consumption	1.193(95% C.I.: 1.025–1.389)	0.023 *	1.015(95% C.I.: 0.881–1.168)	0.841
Length of stay	1.024(95% C.I.: 1.018–1.029)	<0.001 *	1.048(95% C.I.: 1.036–1.061)	<0.001 *
*Enterococcus faecium*	1.57(95% C.I.: 1.148–2.148)	0.005 *	10.545(95% C.I.: 2.665–41.726)	0.001 *
Non-*albicans* Candida in UTIs				
Antimicrobial consumption	1.42(95% C.I.: 1.091–1.848)	0.009 *	0.232(95% C.I.: 0.08–0.673)	0.007 *
Length of stay	1.045(95% C.I.: L1.036–1.053)	<0.001 *	1.045(95% C.I.: 1.033–1.056)	<0.001 *
*Enterococcus faecium* in UTIs	1.665(95% C.I.: 1.173–2.363)	0.004 *	7.347(95% C.I.: 1.522–35.465)	0.013 *

* *p <* 0.05; HAIs: healthcare-associated infections; UTIs: urinary tract infections.

## Data Availability

The data presented in this study are openly available on the following website: https://www.cdc.gov.tw/En/Category/Page/J63NmsvevBg2u3I2qYBenw (accessed on 1 January 2026).

## Supplementary Materials

The following supporting information can be downloaded at: https://www.mdpi.com/article/10.3390/medicina62050814/s1. Supplementary Table S1. The temporal change in the proportion of healthcare-associated infections attributable to Candida species.
