# Supplementary material for: Temporal Trends of Candida Species in Healthcare-Associated Infections in Intensive Care Units in Taiwan
Source: Medicina (Kaunas). 2026 Apr 24;62(5):814. doi: 10.3390/medicina62050814 (PMC13208265; doi:10.3390/medicina62050814)
Supplement: Supplementary file 1 [file medicina-62-00814-s001.zip › medicina-4237299-supplementary.pdf]

Supplementary Table S1. The temporal change in the proportion of healthcare-associated infections attributable to Candida species.

	All Candida	*Candida albicans*	Non-*albicans* Candida
2018-2019	0.42%	0.26%	0.16%
2019-2020	0.01%	-0.26%	0.27%
2020-2021	0.71%	0.24%	0.47%
2021-2022	1.12%	-0.09%	1.2%
2022-2023	-0.64%	0.39%	-1.04%
